# Preterm delivery in Ghana: challenges and implications for maternal mental health trajectories

**DOI:** 10.1371/journal.pone.0317147

**Published:** 2025-03-03

**Authors:** David Kwame Kumador, Alberta Opoku-Mensah, Vivian Tackie-Ofosu, Sheriffa Mahama, Justice Owusu-Bempah, Crossby Osei Tutu

**Affiliations:** Department of Family and Consumer Sciences, University of Ghana, Legon, Accra; York University Faculty of Health, CANADA

## Abstract

**Purpose:**

The present study examined mothers’ experiences with preterm infants in Accra, Ghana, at a time when the COVID-19 pandemic, existing poverty, and global economic depressions severely challenged access to communal, familial, and individual resources. We argue that, in a family crisis, contextual and external institutional resources, such as access to quality healthcare resources, play crucial roles in mothers’ risk exposure and adaptation.

**Study design/methodology/approach:**

Using a qualitative approach with an immersive exploratory-descriptive design, the study interviewed twenty-five (25) mothers whose preterm infants were discharged from the Neonatal Intensive Care Unit (NICU) of Korle Bu Teaching Hospital in Accra, Ghana.

**Findings:**

The study showed that mothers of preterm infants experienced varying range of challenges, including diminished appetite, decreased productivity, and feelings of hopelessness, both during and following their infants’ hospitalization. Having access to adequate income, information, medication, and experienced medical practitioners remains critical to the management of stressful situations associated with the care of preterm children.

**Conclusion for practice:**

Access to funding, preterm information, quality medication, and qualified health professionals can help mothers of preterm infants’ better deal with negative experiences than those who do not have adequate amounts of these resources. Access to critical resources can safeguard mothers’ mental health and the survival of preterm infants within the first year of delivery. A policy on the existing national health insurance scheme can be enacted to expand coverage and absorb the cost of care for the mother and child within the first eighteen months after delivery.

## Introduction

Preterm births represent a significant public health challenge in Ghana, marked by persistent health disparities and barriers to equitable healthcare access [1,2]. They are among the leading causes of neonatal mortality globally, driven by a range of maternal factors such as age, education, and underlying health conditions, including anaemia [2–4]. The mental health implications of preterm births have been highlighted in Ghana, with a large population-based study revealing that mothers of preterm infants face a 79% higher risk of developing mental health challenges compared to their counterparts. This underscores the critical need for targeted psychological support and healthcare interventions to mitigate the associated risks [1,5,6]. Current data indicate a troubling trend: Ghana’s preterm birth prevalence rose sharply from 9.3% in 2006 to 18.3% in 2016, with Northern Ghana experiencing rates as high as 19.4% [2,7].

The impact of preterm births extends beyond neonatal mortality. They pose lasting physical, emotional, and psychological challenges for mothers and children [8]. Preterm infants often face developmental delays, chronic health issues, and higher mortality risks, while mothers grapple with stress, financial strain, and insufficient support [9–11]. Despite the implementation of free maternity care policies in Ghana, systemic inequities persist. Many women encounter cultural barriers, healthcare inaccessibility, and inadequate maternal education, limiting their ability to navigate healthcare systems effectively [12–15].

Globally, approximately 15 million preterm births occur annually, with Africa and Asia contributing 11 million of these. Ghana’s preterm statistics reflect a microcosm of this global burden, necessitating localized interventions [7,16,17]. Research has emphasized the importance of maternal knowledge and community support, highlighting their roles in improving health outcomes for mothers and their preterm infants [18]. However, in Ghana, research has largely focused on clinical outcomes rather than the psychosocial dimensions of maternal experiences, creating a critical knowledge gap [15,19].

Mothers of preterm infants in Ghana often experience intense stress due to uncertain medical outcomes, prolonged hospitalizations, and inadequate healthcare infrastructure [20]. Stress is exacerbated by institutional challenges, including insufficient respectful maternity care (RMC) and cultural insensitivity in healthcare delivery. For instance, RMC initiatives advocate for equitable, dignified, and patient-centred care but remain inconsistently implemented in Ghanaian hospitals [21,22]. Mothers also face challenges transitioning from hospital to home care, further complicating their experiences [11,23].

Social and emotional coping strategies, such as reliance on faith and prayer, have emerged as key mechanisms for Ghanaian mothers managing the challenges of preterm births. These strategies often compensate for the lack of formal psychosocial support systems, particularly in rural and underserved areas [15]. Nevertheless, gaps in healthcare education persist, with many mothers lacking the knowledge and resources to meet the unique needs of their preterm children [9].

The COVID-19 pandemic further exposed vulnerabilities in Ghana’s maternal healthcare system, intensifying the challenges faced by mothers of preterm infants [24–27]. Limited healthcare resources increased financial burdens, and heightened emotional stress underscored the fragility of existing systems [28–31]. Studies emphasize the need for targeted interventions to build maternal resilience and strengthen healthcare accessibility during crises [24,32,33].

This study aims to explore the experiences of mothers caring for preterm infants in Ghana, focusing on the accessibility of healthcare resources and the psychosocial challenges they face. By situating findings within the framework of respectful maternity care, this research seeks to inform interventions that promote maternal well-being and improve outcomes for preterm infants.

## Method

### Study design

The present study employed a qualitative approach with an exploratory-descriptive design [34]. Studies of this nature are often regarded as *sensitive.* The present study was carefully planned and implemented to produce credible results [35]. The study was conducted at the Department of Child Health at the Korle-Bu Teaching Hospital (KBTH) in Accra, Ghana. The Department of Child Health runs speciality clinics, which include neonatal follow-up clinics, on Fridays when mothers of discharged preterm infants are expected to attend these clinics [36]. The department provides a critical setting for studying preterm infants and their mothers’ experiences due to its comprehensive neonatal care capabilities as a reference facility. Research indicates that preterm birth is a leading cause of neonatal mortality in Ghana, with significant rates of complications and deaths observed in the neonatal intensive care unit (NICU) at KBTH, where about 30% of preterm infants die before discharge [37]. Additionally, the hospital’s NICU provides a unique opportunity to explore maternal experiences, as studies have highlighted the psychological and emotional challenges faced by mothers of preterm infants, including financial burdens and the stress of uncertainty regarding their infants’ health [7,38]. Furthermore, the high incidence of neonatal hypothermia and other complications among low-birth-weight infants at KBTH underscores the need for targeted interventions and education, making it an ideal environment for research aimed at improving outcomes for this vulnerable population [39,40].

The study was conducted in 2021. As reported, it was a time when access to social and community resources was severely challenged, and most mothers were experiencing disruptions in mental health because of the challenges that were associated with the COVID-19 pandemic [37,41].

### Participants

A purposive sampling technique was used to recruit 25 mothers for the study. The criteria were that they had delivered a preterm baby, been admitted and discharged from the Neonatal Intensive Care Unit (NICU) and attended a post-NICU clinic for review within the past 18 months. The timeframe was adequate for mothers to recall their experiences and the coping strategies they had employed to mitigate the adverse effects of their experiences [15].

### Instrument for data collection

A semi-structured interview guide with open-ended questions was developed for the study. The guide was informed by literature, theories, and the researchers’ prior experience at the NICU and with families of preterm infants. The interview guide had sections on demographics, some experiences with preterm birth, coping with the baby at home and support systems available to the family. The interview guide was pre-tested with five Greater Accra Regional Hospital (GARH) mothers.

### Data collection procedure

An introductory letter was sent to the Department of Child Health Unit (Neonatal Intensive Care Unit, NICU) to seek permission from the nursing officer and the matron in charge of the NICU clinic. The researchers then introduced themselves to the nurse who oversaw the NICU clinic, and she, in turn, introduced them to the parents with preterm infants. A general overview of the scope of the study was shared with the parents: Mothers who met the predefined inclusion criteria and showed a willingness to participate in the study were recruited with the help of the NICU clinic nurse after they had been informed about the purpose of the survey – consent to participate in the study was sought and signed when mothers agreed to participate.

Mothers who agreed to participate in the research were interviewed face-to-face before they went for their clinical sessions at a designated place at the KBTH hospital, which was assigned to the researchers. Interviews lasted, on average, for 30 minutes. The authors deemed the length of the interviews sufficient, as they were scheduled to accommodate the participants’ availability at the NICU. The setting for the interviews was considered suitable, as it was believed that the sights and sounds of the NICU would aid participants in recalling and effectively sharing their experiences related to the birth and care of their preterm infants.

The participants’ responses were recorded with an audio recorder with their permission.

### Data analysis

Data collection and analysis were done simultaneously to arrive at saturation points in the participants’ responses. The themes identified in earlier interviews were explored in subsequent ones to validate participants’ narratives and probe further into cross-cutting issues that emerge from those narratives. The recorded interviews were transcribed verbatim and back-translated [34] to allow the researchers to analyse the actual, unadulterated responses of the participants – this helped to analyse the intensity and relevance of the issues emerging from the participants. The themes emerging from the data were categorised and re-categorised as the study progressed. Sub-themes were coded and grouped manually. Relevant themes and sub-themes have been presented in Table 1 below and supported with direct participant quotes in the presentation of the study findings.

**Table 1:** Demographic characteristics of participants.

Characteristics	Frequency
Mothers’ Age*	
20-24	1
25-29	4
30-34	9
35 and above	11
Mothers’ Educational Levels*	
Junior High School	4
Senior High School	9
Tertiary	12
Mothers’ Employment Status*	
Not working	2
Self-employed	10
Formal employment	10
Students	3
Number of Other Children*	
1^st^ child	10
2^nd^ Child	6
3^rd^ – nth child	9
Infants’ Gestational Age*	
Extremely preterm ( < 28weeks)	0
Very preterm (28-32 weeks)	16
Moderately preterm (33-36 weeks)	9
Infants’ birth weight**	
Low birth weight ( < 2.5 kg)	15
Very low birth weight (<1.5 kg)	13
Extremely low birth weight ( < 1 kg)	0
Gender of infants**	
Male	13
Female	15

*Total number of mothers who participated in the study =  25.

**Total number of children born to the participants = 28.

### Ethical statement

Ethical clearance was obtained from the University of Ghana’s College of Basic and Applied Sciences Review Board and the Korle Bu Teaching Hospital (KBTH) Institutional Review Board to ensure that the study protocols aligned with procedures for conducting and reporting research with human participants in Ghana (Protocol Number: KBTH-STC 00026/2021). The study was conducted per the Helsinki Declaration. All participants gave informed consent before and during the study. To ensure anonymity, codes (Pn) have been used in this article, where P refers to participants, and n refers to the number of participants. Furthermore, the data (including transcripts and participants’ metadata) was encrypted on a pen drive to ensure that only the researchers could access them. In line with international best practices, the data will be stored securely for fifteen years, after which it will be disposed of.

### Research reflexivity and trustworthiness

Reflexivity and trustworthiness in qualitative research are essential for understanding the interplay between the researchers and the research process, particularly when utilising exclusive qualitative data. They involve continuous self-reflection on how researchers’ values, beliefs, and positionalities shape their interpretations and interactions with participants [42,43]. Reflexivity in qualitative research is essential for ensuring the rigour and trustworthiness of data collection and analysis. For instance, Flogen highlights the challenges researchers face in culturally sensitive environments, where ethical guidelines may conflict with local norms, emphasising the need for transparency in reflexivity [44]. Similarly, Jamie and Rathbone argue that integrating theoretical frameworks during data collection can enhance reflexivity, allowing researchers to navigate their multiple roles and biases effectively [45]. Nicmanis introduces reflexive content analysis as a method that incorporates reflexivity at each analytical stage, ensuring a nuanced understanding of qualitative data [46]. Furthermore, Peddle demonstrates how structured reflexive practices, such as repeated questionnaires, can foster self-awareness and enhance the quality of qualitative research [42]. These insights informed the researchers’ reflexivity in producing credible qualitative findings.

Within the current study context, the research team considered how their interactions with participants might be influenced by their professional backgrounds, experiences, and prior assumptions about preterm infants and their mothers. The researchers/ interviewers were academic researchers from non-clinical backgrounds. The first consideration was determining the participants’ willingness to participate in the study, and providing accurate information to the interview questions could be influenced by the researchers’ backgrounds. With this in mind, the information provided to the participants did not go beyond the researchers’ professional backgrounds. Second, the researchers were mindful not to dominate discussions during the interviews. Therefore, they adopted a backseat approach and allowed the participants to set the pace, hoping they would feel they were in control of their narratives and responses. Third, the notion of *fair dealing* [47] was considered to ensure that no particular participants’ views were represented as the sole truth about the mothers’ experiences. The data analysis consisted of reviewing all transcripts and constantly comparing them to identify similarities and differences without privileging one over the other. This led to the identification of common themes across cases. Fourth, the researchers considered the political and financial implications of their experiences and ensured that the interviews and questions probing did not focus on partisan politics and comparisons between political governments. At the end of each interview, researchers took time to ensure that participants were not distressed by their participation; in these interviews, none of the participants expressed such concerns or appeared distressed or uneasy. Finally, the researchers considered their roles in constructing the participants’ experiences with preterm infants. The research team met frequently to discuss and reflect on the data collection process and the coding of themes. Team members who were not directly involved in the interviewing process took turns reviewing the transcripts and determining whether or not the data was saturated. Two team members who had deeper insights into methodological issues in qualitative studies led the analysis and interpretation of the data.

## Results

The demographic distribution of the participants is presented in Table 1. Eighty per cent of the mothers were at least 30 years old. Mothers within this age range are of advanced maternal age and were seen to be highly at risk of having preterm infants [35]. Research indicates a significant connection between maternal age and the risk of preterm birth, with both very young (<19 years) and older mothers (≥35 years) facing increased risks. A meta-analysis found that while mothers under 19 years had a 1.41 times higher risk of preterm birth, this was not statistically significant [48]. Conversely, mothers over 35 years old are at a notably higher risk due to factors such as underlying health conditions and increased likelihood of multiple pregnancies, which can lead to complications [49]. Additionally, advanced maternal age (≥45 years) significantly raises the risk of preterm birth, particularly in pregnancies resulting from in vitro fertilisation [50]. A pan-Nordic study further corroborated these findings, showing that maternal ages of 38-39 years are associated with a 2.1 to 2.4 percentage point increase in preterm delivery risk compared to ages 26-27 [51]. Thus, maternal age is a critical factor influencing preterm birth outcomes.

The preterm infants in the present study were categorised according to the World Health Organisation’s measures of gestation before delivery [7]: moderately preterm (33 to 36 weeks), very preterm (28 to 35 weeks), and highly preterm (28 weeks and below). Thus, the deliveries in the present study were considered to vary from moderate to very preterm, with all the infants having low birth weights. Fifteen infants were classified as having low birth weight (2,500 g), while 13 had very low birth weight (less than 1,500 g). Two of the 25 mothers interviewed had multiple births [7]^,^ while the rest had single births. Further, fifteen of the mothers have had other children before.

### Experiences during hospitalisation

It was found in the present study that most infants relied on life support machines for survival due to their underdeveloped respiratory systems. The infants labelled as high-risk newborns, especially the very preterm ones, were vulnerable to infections. They were, therefore, restricted to incubators with respiratory and nutritional support systems. The participants reported that seeing their children on this life support system stressed them so severely. A participant shared her experiences with the researcher by narrating a scene at the ward that left her traumatised for days:

*I saw it with my eyes how a baby lying close to my [twin] kids died, and they wrapped him, and then the next day another baby died again…. I was going mad, [I said to myself repeatedly] what will happen to my infants? (P3, 31 years old, 31 weeks preterm babies, emphasis added).*

These and other similar experiences faced by the participants were further exacerbated by the limited time they had to prepare adequately. Ten mothers expressed how the unexpected delivery disrupted their lives and livelihoods. According to the mothers, there were sudden disruptions in their day-to-day activities, for those engaged in the informal sector as petty traders had their significant sources of livelihood severed during the time they spent at the hospital and home after the babies were discharged. Having no regular money and means of generating income affected them economically and psychologically. A mother elucidated this further, stating:

*I just woke up one Thursday feeling some pains, abdominal pains, thinking that it was normal pain, so I needed to see the doctor for medications. I just took paracetamol at home, and then, on Friday morning, the pains were still severe. I needed to come to the hospital. When they examined me, they said it was labour…? I had to call my family for them to bring my luggage and things because I was going to the labour ward… I did not feel happy because my time was not due* (P3, 31 years, 31 weeks preterm baby).

For some of the participants, their expectation of giving birth and going back home with healthy babies within three days after delivery or a week if they went through a caesarean section was thwarted. The event’s suddenness made them feel “disorganised” (P16, 31 years, 30 weeks preterm baby). The traumatic experiences that the participants encountered were further amplified by the reactions of the doctors and family members when they delivered preterm babies. For some mothers, the traumatic experiences were further triggered by the responses of family members to the birth of the preterm child:

*When I was even here, some [family members] told me to come home since the baby would not survive* (P8, 37 years, 30 weeks preterm baby).

### Experiences after hospitalisation

Another theme identified in the study was mothers’ post-NICU experiences. These were experiences of mothers caring for their preterm infants at home after they had been discharged from the intensive care unit. A key challenge for the mothers was having their children readmitted multiple times. According to some of the mothers, the preterm infants were vulnerable to infections due to their immature physiology, resulting in numerous readmissions at the clinic.

*At three months, she ran a temperature, and the doctor said it was an infection, so she was admitted for one week*” (P17, 38 years, 36 weeks preterm baby).

*At first, she fell sick and was admitted to ER [Emergency Room], later she fell ill again and was admitted to Child Health…there was an infection”* (P18, 32 years, 36 weeks preterm baby).

Beyond multiple readmissions, the mothers had to deal with the stigma that came with the size and physical appearance of the infant after she was discharged.

*Our last born told me to go and throw the baby away because she was too tiny. I even lost hope”* (P12, 36 years, 32 weeks preterm baby).

In Ghanaian cultures, infants were expected to look “fleshy and weighty” [52]. Having ‘tiny’ children made them feel worried and ashamed. Three of the mothers said they had to hide the children for six months before performing the naming ceremony.

*We felt embarrassed to let people come and look at them, and they felt like we were telling them they were witches or something* (P3, 31 years, 31 weeks).

*When I was coming for review, I would wrap him tightly…I felt shy because I hadn’t given birth to a baby like that before* (P13, 39 years, 32 weeks).

*Because she was very tiny, we had the naming ceremony three weeks ago, and she is seven months old now* (P12, 36 years, 32 weeks).

The thought of having tiny babies affected the mothers’ mental health as well. A participant shared how she was affected psychologically by her baby’s size:

*When they are very tiny, sometimes you hold the baby, and when something falls off your hand, you presume that the baby has fallen because they are very tiny. Assuming my handkerchief falls, there is that kind of panic, thinking I have dropped the baby fall* (14, 38 years, 28 weeks, emphasis added).

The experiences of the mothers were heightened by their inability to access adequate social support. Two of the mothers explained this further, stating:

*I didn’t have anyone to help, so I suffered. I do everything by myself, especially when having bodily pains and needing to sleep, but the baby is crying; you must pick her up* (P11, 35 years, 32 weeks).

*My husband is often away from home, so I do house chores. Then, I must take care of her alone, which is stressful for me* (18, 32 years, 36 weeks).

Previous studies (Eriksson, 2022) suggest that social support is critical to overcoming emotional stress. Thus, the mothers in the present study had increased stress because they received little or no support from relatives and friends other than their spouses.

*It affects productivity because sometimes she cries. She wants to eat, so I must take her and feed her before I continue whatever I am doing – so it reduces my productivity at work* (P18, 32 years, 36 weeks preterm).

For mothers who were in formal employment, their productivity was severely affected as a result. In some cases, they were compelled to take the babies to work because they were too young, tiny, and fragile to send to daycare, even if they could afford such a facility.

### Stress coping strategies

#### Taking leave from work activities.

Taking time off work to adjust to the unexpected routine provided ample time to care for their babies. Some mothers either took extended maternal leave, sought more flexible jobs, or quit altogether to stay home.

*Time has helped me in a way. In my previous work, I had to leave home early in the morning and return after 9 p.m. I used to be a teller at the bank, and I had to wait and balance the accounts for the day before leaving the bank. I don’t know what I would have done if I was still working. I resigned in December 2019 and gave birth in 2020. I don’t see how it would have been, how long the [employer) was going to give me a leave advantage. It would not be possible, and they are tiny. If it were a normal baby, three months, I would have to resume work. So, I think being off my job for a while was necessary to take care of him, and because of that, I can rest at times* (P14, Banker, 38 years, 28 weeks preterm).

Although that worsened their financial conditions, it inevitably gave them ample time to deal with the stressful situations of caring for the preterm and safeguarding their mental health.

**Positive thinking.** Reflecting more on their positive experiences was a critical strategy the mothers employed to navigate their way. Two mothers pointed out that relying on inner strengths and thinking positively was instrumental to coping with the challenges of having a preterm baby.

*The joy I had gave me the strength to do everything. I do everything* (P6, 46 years, 32 weeks preterm).

*I am somebody who doesn’t give up; it strengthens me, and I just keep going* (P17, 38 years, 36 weeks preterm).

Several psychosocial theories on coherence, hardiness, self-efficacy, and optimism point to the power of a positive mindset in helping people endure hardships and stressful situations [52–55].

#### Access to adequate healthcare resources.

Having enough money to cater for the family and the newborn baby and having access to the correct information and medication were important coping resources for the mothers. The presence of adequate funds made coping with preterm parenting less stressful, as the mothers were able to meet various needs. This was highlighted by three mothers as follows:

*There are a lot of costs, and if you don’t have enough money, you suffer” *(P8, 37 years, 30 weeks preterm).

*If I didn’t have money, the baby might not have survived. He was super small, tiny. At the NICU, it was not easy, and you were doing labs and buying drugs, always doing labs and buying drugs *(P13, 39 years, 32 weeks preterm).

*We were buying everything, yet we had insurance, which has helped” *(P14, 38 years, 28 weeks preterm).

As reported elsewhere, mothers who made financial plans were more likely to cope well with challenges in the future [51,52]. Access to information on how to care for preterm babies helped the mothers learn more about their situations and derive inspiration from others who had successfully navigated caring for preterm babies. Some mothers searched for information online, while others used instructions and information given before discharge. Out of the 25 mothers interviewed, 18 were first-time mothers. These mothers narrated how important it was for them to rely on information from doctors, friends, social media, and the internet. The following are excerpts from three of the mothers.

*I usually search the internet for what to do at what time* (P14, 38 years, 28 weeks preterm).

*The doctors spoke to us and gave us information, so I followed their advice, and it helped me immensely* (P1, 31 years, 34 weeks preterm).

We found a couple of videos on YouTube and some apps I cannot recall the names of right now that guide us on how to handle them, feed them…. know the acceptable temperature, change in complexion, and what it means (P19, 29 years, 31 weeks preterm).

To the mothers, having the proper medication contributed significantly to their preterm infants’ development as they saw weight gains and improvement in the global well-being of the preterm infants after they were administered the drugs.

*The medicine they gave her was beneficial* (P18, 32 years, 36 weeks preterm).

*Some medications were given to them, so they ate more and were eager to eat whatever we fed them. Although she looked tiny earlier, the medicines helped her develop faster* (P3, 31 years, 31 weeks preterm).

#### Interactions with health professionals.

The study participants derived some encouragement from their interactions with medical professionals at the NICU.

*The counsellor called me and asked what I had heard about my child… and she said she would be fine and that I should not cry because if I cry, the baby becomes sorrowful* (P10, 30 years, 33 weeks preterm).

*The doctors spoke to us and gave us information, which helped me immensely* (P1, 31 years, 34 weeks).

This resource, though intangible, has been shown to help mothers with preterm infants to cope with the stress [56,57]. Through their interactions at the clinic, they have acquired valuable and adequate information on concepts such as childcare, social stigmatisation, and self-worth.

## Discussion

Three themes were identified, namely, mothers’ experiences during hospitalisation, mothers’ post-NICU experiences, and healthcare and coping resources available to mothers. These emergent themes are consistent with other studies [57,58] that reported that most maternal experiences with preterm birth were challenging and negative. The study’s findings are discussed using the family resiliency theory [59]. Walsh [59] elucidates resilience as the individual/familial capacity to bounce back from a crisis using available protective factors. We argue that in the absence of family protective factors instantiated by limited family resources and stigma towards preterm infants, mothers seek external protective factors as scaffolds to navigate crises and safeguard their mental health. The trajectories of maternal mental health in response to negative experiences of preterm delivery and care were mediated by the availability/unavailability of familial resources. There is sufficient evidence from the findings of the present study to suggest that mothers’ experiences, which initially emerged as natural responses to the ‘unexpected’ crisis of preterm delivery, were mediated by factors such as stigma, lack of social support systems, unplanned expenses and financial burden, and fears of losing the child. The presence and quantum of these factors have consequences for the mental health trajectories of the mothers and the survival of the child. Experiences with social stigma, isolation, and inadequate financial resources tend to destabilise the mothers’ intrapsychic balance, leading to adverse outcomes of stress, depression, and hopelessness. This assertion is supported by similar studies [60,61] that report associations between adverse experiences and psychosocial disorders such as stress and depression.

However, in some mothers, the effects of the negative experiences were mediated by having access to quality healthcare resources. The findings suggest that the mothers who had access to quality information, medication, and qualified health professionals, perhaps due to their financial status, managed negative experiences better than those who needed adequate resources. Thus, the mothers, especially first-time mothers, greatly benefited from these resources. Such mothers employed these resources as scaffolds or shores to navigate the crisis associated with preterm delivery. The healthcare resource consisted of accessing information and medication through professional medical counselling. Maternal resilience, moderated by healthcare resources, emerges as a critical psychosocial artefact in crisis management about preterm delivery and care. Subsequently, maternal resilience led to lower rates of infections and less frequent readmissions of their children. Studies show that coping during childcare is based mainly on the amount and nature of information available to parents [57,59].

Furthermore, the study particularly highlighted the significance of metaphysical coping resources, such as one’s belief in the supernatural, as a mediating factor in the care of preterm infants and the regulation of maternal stress. In cases where familial, social, and personal risk protective factors were inadequate to counter maternal negative experiences, belief in the supernatural became an intervening factor between stress and positive thinking, of hopelessness and encouragement.

### Conclusion for practice

The study showed that mothers of preterm infants go through stressful situations as they attend postnatal clinics to care for their children. The study further demonstrates that preterm care can have far-reaching consequences for the mental health of mothers, their productivity at work and livelihoods, and the survival of the children they care for. However, having access to adequate healthcare resources in terms of information, medication, and experienced medical practitioners remains critical in minimising maternal stress. The findings of the study have broader implications for policy in maternal care and mental health in Ghana. First, the National Health Insurance Scheme and the Ghana Under-five Infant Health policies can be reviewed to allow mothers of preterm infants to access unlimited quality healthcare services at no cost – for at least the first six months after delivery. This will substantially reduce the financial burden on mothers while ensuring their mental health and general well-being are sustained. Second, institutionalising respectful maternal care (RMC) programmes in hospitals and clinics will help provide tailor-made resources to mothers and families during and after preterm delivery to minimise stress and safeguard their mental dignity. Third, continuous mass education systems on possible causes, prevention, and care for preterm children should be strengthened to address social stigma and garner communal and familial support for mothers caring for preterm children. Finally, a legislative instrument can allow fathers of preterm children to access up to three months of paid leave or flexible working conditions to support their spouses and preterm children adequately.

## Limitations of the study

Some limitations of the study were identified. The study is limited to the recalled experiences of mothers of preterm infants, specifically those who attended the Neonatal Intensive Care Unit of the Korle Bu Teaching Hospital in Accra, Ghana. Thus, the responses may not be representative of all mothers with preterm infants. The data was collected after the COVID-19 pandemic; therefore, it is assumed that experiences with COVID-19 could have conflagrated the participants’ narratives.
